# Improving preventive service delivery at adult complete health check-ups: the Preventive health Evidence-based Recommendation Form (PERFORM) cluster randomized controlled trial

**DOI:** 10.1186/1471-2296-7-44

**Published:** 2006-07-12

**Authors:** Vinita Dubey, Roy Mathew, Karl Iglar, Rahim Moineddin, Richard Glazier

**Affiliations:** 1 Dept of Public Health Sciences, University of Toronto; 1 Bluenose Cres, Toronto ON M1C 4R7, Canada; 2 Dept of Family and Community Medicine, St. Michael's Hospital, 30 Bond St, Toronto ON M5B 1W8, Canada; 3 Inner City Health Research Unit, University of Toronto and St. Michael's Hospital, 30 Bond St, Toronto ON M5B 1W8, Canada

## Abstract

**Background:**

To determine the effectiveness of a single checklist reminder form to improve the delivery of preventive health services at adult health check-ups in a family practice setting.

**Methods:**

A prospective cluster randomized controlled trial was conducted at four urban family practice clinics among 38 primary care physicians affiliated with the University of Toronto. Preventive Care Checklist Forms^© ^were created to be used by family physicians at adult health check-ups over a five-month period. The sex-specific forms incorporate evidence-based recommendations on preventive health services and documentation space for routine procedures such as physical examination. The forms were used in two intervention clinics and two control clinics. Rates and relative risks (RR) of the performance of 13 preventive health maneuvers at baseline and post-intervention and the percentage of up-to-date preventive health services delivered per patient were compared between the two groups.

**Results:**

Randomly-selected charts were reviewed at baseline (n = 509) and post-intervention (n = 608). Baseline rates for provision of preventive health services ranged from 3% (fecal occult blood testing) to 93% (blood pressure measurement), similar to other settings. The percentage of up-to-date preventive health services delivered per patient at the end of the intervention was 48.9% in the control group and 71.7% in the intervention group. This is an overall 22.8% absolute increase (p = 0.0001), and 46.6% relative increase in the delivery of preventive health services per patient in the intervention group compared to controls. Eight of thirteen preventive health services showed a statistically significant change (p < 0.05) in favor of the intervention (adjusted RR (95% C.I.)): counseling on brushing/flossing teeth (9.2 (4.3–19.6)), folic acid counseling (7.5 (2.7–20.8)), fecal occult blood testing (6.7 (1.9–24.1)), smoking cessation counseling (3.9 (2.2–7.2)), tetanus immunization (3.0 (1.7–5.2)), history of alcohol intake (1.33 (1.2–1.5)), history of smoking habits (1.28 (1.2–1.4)) and blood pressure measurement (1.05 (1.00–1.10)).

**Conclusion:**

This simple, low cost, clinically relevant intervention improves the delivery of preventive health services by prompting physicians of evidence-based recommendations in a checklist format that incorporates existing practice patterns. Periodic updates of the Preventive Care Checklist Forms^© ^will allow a feasible and easy-to-use tool for primary care physicians to provide evidence-based preventive health services to adults at routine health check-ups. The forms can also be incorporated into an electronic health record. The Preventive Care Checklist Forms^© ^are accessible in English and French at the College of Family Physicians of Canada web site.

## Background

Integration of preventive care guidelines into clinical practice has been modest across all medical specialties and countries [1-13]. Reasons cited for why physicians do not follow clinical practice guidelines include a lack of awareness of guidelines, lack of familiarity to apply guidelines correctly, lack of agreement with the guidelines, lack of a believe that physician behaviours or outcomes can be changed and external barriers such as a lack of time or a reminder system [14-16].

The Canadian Task Force on Preventive Health Care grades preventive health recommendations into five categories from A to E based on the strength of evidence for a specific preventive health maneuver, similar to the US Preventive Services Task Force and other such organizations [17,18]. Grade A recommendations have good evidence to include in adult preventive service delivery, usually from randomized controlled trials, and B recommendations have fair evidence to include. Examples of grade A recommendations include colorectal cancer screening with a fecal occult blood testing, prevention of neural tube defects through folic acid supplementation, and providing smoking cessation counseling; and grade B recommendations include cervical cancer screening with a Papanicolaou smear and ensuring rubella immunity. The Task Force recommendations are most applicable to primary care physicians who conduct health examinations on asymptomatic individuals and have the greatest opportunity for prevention.

Primary care physicians self-report excellent delivery of preventive services but in reality rates are moderate to low [19-23]. In one study of 4, 049 patient visits in 138 family physician offices in Ohio, for three groupings of preventive service delivery, patients were up to date on only 55% of screening, 24% of immunization and 9% of health habit counseling services [24]. Cancer screening has shown similar poor outcomes in primary care, despite the evidence that cancer screening can reduce morbidity and mortality when provided to populations of patients [17,18,25-27]. Even at adult health check-ups, a time typically devoted to providing preventive health care, rates of preventive service delivery are low [24].

Barriers to the delivery of preventive health services include lack of time, physician forgetfulness, patient refusal, practice logistical difficulties, urgent concerns dominating visits, inadequate reimbursement and patient concerns regarding the intervention [28-30]. It is estimated that 7.4 hours per working day are required just for the provision of preventive services by a primary care physician [31]. Tools to improve prevention must overcome such barriers [32,33]. Otherwise, as Stange *et al *argue, it may be unrealistic to expect primary care physicians to deliver a comprehensive package of clinical preventive services among the many competing demands of providing primary care [24]. However, if primary care physicians are unequipped to deliver preventive health services, then how can patients and populations hope to receive these services? With an aging population the ability of a health care system to focus on prevention is even more essential to reduce preventive morbidity and mortality.

Continuing medical education and continuing professional development have been the traditional modalities for implementing changes in clinical practice, but their effectiveness has been disappointing. Knowledge translation is a new paradigm for putting knowledge into practice by incorporating tools to overcome barriers to change [34-36]. Prompts, reminders and patient-mediated methods are examples of such tools.

There is no one proven maneuver to improve preventive care [5,37,38]. Multi-faceted interventions especially with organizational change are valuable, yet they are expensive, logistically complex to implement and are not consistently more effective than single interventions. Other successful interventions are not easily incorporated into routine practices as they require a nurse facilitator, complex computer systems, office-system changes and "teamwork and tenacity" [39,40]. More research has been advocated to determine methods to disseminate prevention guidelines into practice.

Prompting physicians can lead to a significant improvement in health maintenance, such as checklists attached to the patient chart, tagged notes, computer generated encounter forms, prompting stickers and patient carried prompting cards [32]. Studies on physician reminders are dated, though they did consistently show improved rates of preventive activities between 6% and 24% compared to a control group [10,38]. Computer generated reminder systems have been popular, but are expensive and require advanced computerized office systems, yet only 5 to 13% of primary care physicians have electronic health record systems.

Are simple reminder tools, such as a checklist form, still an effective way to assist providers to adopt preventive health care guidelines into daily practice? A few studies done in the 1980's consistently showed that checklists were highly adopted, continued to be used months after the commencement of an intervention, and were effective to increase rates of preventive service delivery [41-45]. Recently, Litaker *et al *have urged that future interventions should address visit- and practice-specific factors that are more closely associated with preventive care [46]. In this study, we provide primary care physicians with a evidence-based, sex-specific Preventive Care Checklist Form^©^, to be used at adult health check-ups, to determine if this visit- and practice-specific tool will be effective to improve the delivery of preventive health services.

## Methods

### Setting

The trial was conducted at four academic family medicine clinics affiliated with St. Michael's Hospital and the University of Toronto. Over 40 family physicians and 20 residents see approximately 100 000 patient visits per year. The four clinics are part of the same hospital department that share clinical guidelines and practices, educational rounds, staffing levels, hours of operation, after-hours care, and teaching duties. Each clinic has diverse patient populations including traditional family practice and marginalized groups including those with low-income, disability, HIV/AIDS, and severe and persistent mental illness. Each clinic has the same access to health care and preventive services.

### The intervention

Male and female evidence-based Preventive Care Checklist Forms^© ^were developed using Canadian Task Force on Preventive Health Care recommendations and other sources where the Task Force had no up-to-date guidelines [see Additional File 1 and 2]. Grade A (good evidence to include) or B (fair evidence to include) recommendations were delineated by bold and italics text respectively. Non-evidence based but practice relevant components including functional inquiry and general physical examination were added. Male and female forms were photocopied on blue and pink paper respectively. An explanation sheet detailing the recommendations accompanied the form [see Additional File 3]. Pilot testing was conducted on 10 unaffiliated family physicians.

Stratified randomization by clinic size was used since two of the four clinics are large and the other two smaller and community-based. Clinics were randomized using a random number table. CONSORT guidelines for cluster randomized controlled trials were adhered to [47,48].

All participating physicians gave informed consent for a trial on preventive medicine. No other details of the study were disclosed. In the control clinics, usual preventive care did not include any structured or organizational components. In the intervention clinics, physicians, nurses and clerical staff were informed at staff meetings, by email and in person that Preventive Care Checklist Forms^© ^were available. They were not aware that the forms were part of a study or if the forms were to be evaluated. Sex-specific forms were attached by clerical staff to the chart of adults booked for complete health check-ups, which are usually 20–30 minute scheduled appointments. No other additions or restructuring of preventive services took place during the study period. Completed forms acted as documentation of the visit and were filed in the patient's chart. The forms were implemented in the intervention group during a 5-month period from November 2002 to March 2003. The study was approved by St. Michael's Hospital Research Ethics Board, Toronto, Canada.

### Data collection

A simple stratified random sampling technique generated lists from the insurance billing database of adult patients who had a complete health check-up, of equal proportion per physician, from January to June 2002. Baseline characteristics of the patient and preventive health services offered to the patient were recorded on a standardized form by two chart abstractors. Chart abstractors were graduate students in epidemiology, trained using standard methods. Abstractors were blinded in the period before the intervention. The presence of the form precluded blinding in the post-intervention period. An identical chart review was carried out using a similar sampling technique for the post-intervention period from November 2002 to March 2003. A summary of physician practice profiles and physician characteristics was determined using the hospital and insurance billing databases averaged over the study period.

Thirteen pre-selected preventive maneuvers were recorded out of a possibility of 41 A and B evidence-based recommendations. The maneuvers selected were a mixture of preventive health services known to be well (blood pressure, clinical breast exam, Pap smear and smoking history), moderately (alcohol history, brushing/flossing teeth, hearing assessment, mammogram and smoking cessation) and poorly (folic acid supplementation, fecal occult blood testing, rubella immunity and tetanus immunization) implemented in our setting based on continuous quality improvement assessments, and rates found in other settings [20,21,24]. Aside from the study investigators, the clinics were not aware how or which preventive maneuvers, if any, were to be evaluated.

The whole chart, including requisitions, laboratory reports and consult notes, was reviewed to determine rates of the thirteen preventive maneuvers. A maneuver was considered complete if either the patient received or was offered yet refused it. Maneuvers not documented were considered not performed. Charts were excluded if the patient was younger than 21, an investigator completed the assessment or the encounter was disease/illness specific. To determine if a preventive health service was provided to a patient, age and/or sex specific criteria had to be met based on the criteria for each preventive health maneuver from the Canadian Task Force on Preventive Health Care [17]. The following exclusions were applied only to the eligibility of a patient to receive a particular preventive health service; the whole patient chart was not excluded. Women 21 to 45 years were excluded from folic acid supplementation and rubella immunity if they had a hysterectomy, bilateral oopherectomy, tubal ligation or confirmed menopause. Women 21 to 69 years were excluded from a Pap smear if they had a total abdominal hysterectomy, cervix removed or were never sexually active. Eligible women were excluded from a screening breast exam and mammogram if they had a previous history of breast cancer. Adults older than 50 years were excluded from fecal occult blood testing if they had a history of colon cancer or a sigmoidscopy/colonoscopy in the past 5 years. Seniors were excluded from a hearing assessment if they were known to be deaf.

### Analysis

The sample size was calculated to detect a mean difference of 30%. For the sample size calculation, consistent with other studies, we assumed a conservative estimates of a 50% prevalence for each maneuver at baseline, an ICC of 0.02, a 5% type I error, and 85% power [49,50]. Two-hundred twenty charts in each of the control and intervention group were needed to detect a 30% change. Using an alpha type I error of 0.05 and 90% power, 273 charts were required per group.

Data were input into SPSS version 11 and analyzed using SAS 8.2. An intention-to-treat analysis was used for form utilization. No forms were found in the control site.

The overall summary outcome was the percentage of up-to-date preventive health services delivered per patient between the two groups, computed using a t-test. The rates and relative risks of the performance of the 13 preventive health maneuvers were the trial endpoints. In a univariate analysis, if a potential confounder was significant (p < 0.10) for a single maneuver it was retained in the final model for all maneuvers. Adjusted odds ratios were calculated in a separate model for each maneuver using Poisson regression with log link, controlling for cluster randomization, pre-intervention rate of the maneuver for that group, the number of years in practice of the physician, average proportion of patients seen per half-day, average number of visits per patient, Charlson comorbidity scale [51], and mental health diagnosis. The Charlson comorbidty scale and mental health diagnosis together were used as a proxy of patient complexity and comorbidity in this ambulatory care setting. Logistic regression was not used since preventive outcomes were not rare, and odds ratios would over-estimate the true relative risks. With Poisson regression, the adjusted relative risks could be estimated [52]. All outcomes were controlled for cluster randomization.

## Results

A total of 38 of 42 physicians were involved in the trial (90.4%), 20 in the intervention arm, 18 in the control. Three physicians were excluded because they were involved in the study and one did not have an active practice. The adult health check-up was billed in 7.7% of all ambulatory encounters, and 33.6% of patients had at least one health check-up billed in 2002.

**Figure 1:**
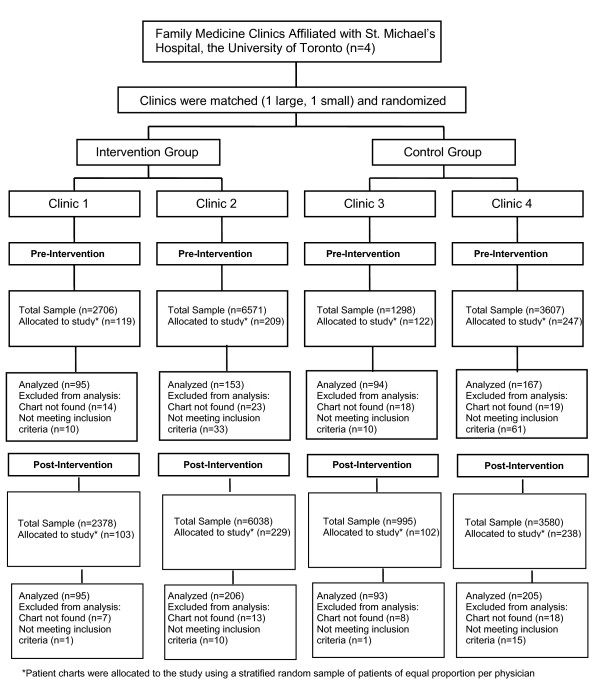
Randomization process and progress through the study.

At baseline, a total of 509 patient charts were included in the study (Figure 1). Table 1 lists baseline characteristics of patients, physicians and physician practice profiles. Compared with the control arm, the intervention arm had a higher proportion of female patients, less comorbidity, fewer patients with a mental health diagnosis and physicians were somewhat older, in practice longer and had more health check-ups per patient visit. Baseline data on education, occupation, income, language and ethnicity were not included because of poor chart documentation.

**Table 1:** Characteristics of patients, physicians, and physician practice profiles at baseline assessment

**Patient Characteristics**:	**Intervention n (%)**	**Control n (%)**
Sample Size	248	261
Mean Age (SD)	47.0 (16.5)	41.9 (14.1)
Female	160(64.5)	148(56.7)
Married/Common-law	91(40.1)	74(33.5)
Charlson Comorbidity Scale*		
0–2	240(96.7)	233(89.3)
3–8	8(3.3)	28(10.7)
Mental Health Diagnosis	25(10.2)	52(20.1)
Number of visits at Clinic		
≤3 visits	62(25.1)	53(20.9)
4–9 visits	40(16.2)	53(20.9)
≥10 visits	145(58.7)	148(58.2)
Resident Involved	16(6.6)	28(10.7)
		
**Physician Characteristics**:		
Sample Size	20	18
Mean Age (SD)	45.9 (10.1)	37.8 (7.3)
Female Sex	11(55.0)	11(61.1)
Mean Number of Years in Practice (SD)	16.6 (9.1)	7.5 (6.2)
		
**Physician Practice Profiles**:		
Mean Age of Patients (SD)	46.5 (4.4)	43.9 (3.0)
Females in Practice	(63)	(48.9)
Complete Health Check-ups per Patient Visits	(10.0)	(5.8)
Mean Half-Days per Week (SD)	5.5 (2.8)	5.6 (1.3)
Mean Patient Seen per Clinic Day (SD)	14.0 (7.0)	13.7 (3.6)
Mean Patient Visits per Patient (SD)	3.0 (0.5)	3.0 (0.7)

*Charlson ME, Pompei P, Ales KL, McKenzie CR. A new method of classifying prognostic comorbidity in longitudinal studies: Development and validation. J Chron Dis 1987;40:373–383.

Inter-observer reliability was high with a kappa of 0.80 (range 0.70 to 0.97). For coders A and B, the intra-observer reliability was κ = 0.94 (range 0.80 to 1.0) and κ = 0.87 (range 0.64 to 0.97) respectively.

During the post-intervention period, a total of 608 patient charts were analyzed. More patients were included compared to baseline because of fewer chart exclusions. The rates of preventive health maneuvers at baseline and post-intervention in both groups are listed in Table 2. At baseline, there were no significant differences between the intervention and control group with the exception of brushing/flossing teeth (p < 0.05). However, after controlling for physician practice profiles (mean age of patients, number of health check-ups per patient visits, mean half-days per week, mean patients seen per clinic day and mean patient visits per patient) differences in all 13 maneuvers became non-significant at baseline. Rates varied from 3% for fecal occult blood testing to 93% for blood pressure measurement.

**Table 2:** Rates of preventive maneuvers performed at baseline and post-Intervention in both Groups

	**Intervention Group**				**Control Group**			
	
	**Baseline**	**Post-Intervention**	**Change**	**P-value**	**Baseline**	**Post-Intervention**	**Change**	**P-value**
	
**Preventive Maneuver**	**n (%)**	**n (%)**	**%**		**n (%)**	**n (%)**	**%**	
Brushing/Flossing	174 (29.8)	310 (47.9)	18.1	<0.001	239 (8.4)	298 (6.7)	-1.7	0.51
Blood Pressure	246 (93.5)	310 (96.6)	3.1	0.11	259 (92.7)	298 (93.0)	0.3	0.89
History of Alcohol	248 (66.5)	310 (90.9)	24.4	<0.001	261 (74.7)	298 (74.8)	0.1	0.97
History of Smoking	248 (73.4)	310 (92.4)	19	<0.001	261 (79.7)	298 (79.2)	-0.5	0.91
Smoking Cessation	43 (39.5)	45 (70.0)	30.5	0.005	78 (29.5)	71 (25.4)	-4.1	0.58
Tetanus Immunization	242 (12.8)	308 (40.9)	28.1	<0.001	259 (19.7)	297 (9.4)	-10.3	<0.001
Folic Acid Counseling	96 (8.2)	119 (34.7)	26.5	<0.001	111 (10.9)	125 (3.2)	-7.7	0.03
Rubella Immunity	96 (15.5)	119 (34.7)	19.2	0.002	111 (10.0)	125 (9.6)	-0.4	0.93
Breast Exam	157 (82.1)	173 (97.4)	15.3	<0.001	149 (80.7)	148 (77.7)	-3	0.57
Mammography	55 (41.8)	50 (76.6)	34.8	<0.001	33 (57.5)	16 (50.0)	-7.5	0.76
								
Pap Smear	148 (73.6)	164 (84.7)	11.1	0.02	145 (86.2)	145 (88.3)	2.1	0.72
Fecal occult Blood	83 (13.4)	102 (50.6)	37.2	<0.001	55 (3.6)	67 (7.5)	3.9	0.45
Hearing Assessment	44 (31.8)	44 (41.2)	9.4	0.50	22 (22.7)	33 (12.1)	-10.6	0.45

The rate of form utilization was 84% in the intervention group. In the unadjusted intention-to-treat analysis during the post-intervention period, 11 of 13 maneuvers were statistically significant, all in support of the intervention (Table 2). After adjustment for confounders, 8 of 13 remained significant and rubella immunity, clinical breast exam and hearing assessment became non-significant, while blood pressure measurement reached significance (Table 3).

**Table 3:** Unadjusted* and adjusted^† ^relative risk of performance of preventive health maneuvers post-intervention

**Preventive Maneuver**	**Sample Size**	Unadjusted* Relative Risk (95% CI)		**Adjusted† Relative Risk (95% CI)**	
Brushing/Flossing	605	6.96	(3.93–10.07)	9.19	(4.32–19.57)
Blood Pressure	605	1.03	(0.98–1.09)	1.05	(1.00–1.10)
History of Alcohol	605	1.23	(1.05–1.45)	1.33	(1.17–1.51)
History of Smoking	605	1.20	(1.05–1.37)	1.28	(1.16–1.42)
Smoking Cessation	116	2.55	(1.60–4.06)	3.93	(2.16–7.15)
Tetanus Immunization	605	4.35	(2.37–7.98)	3.00	(1.72–5.22)
Folic Acid Counseling	244	10.20	(4.36–23.90)	7.47	(2.69–20.75)
Rubella Immunity	244	3.43	(1.32–8.89)	3.14	(0.78–12.62)
Breast Exam	321	1.25	(1.09–1.43)	1.06	(0.97–1.16)
Mammography	66	1.49	(0.90–2.48)	1.41	(0.76–2.61)
Pap Smear	309	0.96	(0.86–1.07)	0.92‡	(0.83–1.01)
Fecal occult Blood	169	4.96	(1.81–13.55)	6.69	(1.85–24.17)
Hearing Assessment	77	9.50	(1.46–61.87)	5.13	(0.70–37.32)

*Controlled only for Cluster Randomization.

†Poisson regression (continuous variable unless otherwise stated) controlling at the level of the clinic: cluster randomization and the pre-intervention rate for that group; at the level of the physician: the number of years in practice; at the level of the practice demographics: mean proportion of patients seen per half-day and average number of visits per patient; and at the level of the patient: mean age, comorbidity (categories of 0, 1, 2), and mental health diagnosis (categories of Y, N).

‡For model stability, unable to adjust for mean patient age.

The overall summary statistic was a comparison of the percentage of up-to-date preventive health services delivered per patient in each group (Table 4). Before the intervention, each patient received on average 51.8% of maneuvers in the control group and 51.4% in the intervention group (p = 0.81). After the intervention, the results were significantly different (p = 0.0001). In the control group each patient was up-to-date on only 48.9% of eligible maneuvers, while the intervention group was up-to-date on 71.7% of eligible maneuvers. This is an absolute difference of 22.8% between the two groups, corresponding with a relative increase in preventive care of 46.6%.

**Table 4:** Summary outcome: percentage of up-to-date preventive health services delivered per patient at baseline and post-intervention

**Before Intervention**	**Sample Size**	**Mean %**	**95% CI**
Control Group	261	51.8	(49.8–53.9)
Intervention Group	248	51.4	(48.6–54.2)

**After Intervention**			

Control Group	298	48.9	(47.0–50.8)
Intervention Group	310	71.7	(65.1–78.3)

## Discussion

This study evaluates a single double-sided checklist reminder form to be used by physicians at adult health check-ups to improve a broad range of preventive health services. The results of this study strongly support the effectiveness of the Preventive Care Checklist Forms^© ^to improve the delivery of preventive health care. This simple intervention resulted in a clinically important, 22.8% absolute change in preventive care, of larger magnitude than many multi-faceted complex prevention trials, where an absolute increase of 10% is usually considered an excellent outcome.

This study makes a significant contribution beyond prior work in this area for several reasons. Through this study, the Preventive Care Checklist Forms^© ^have been validated and endorsed by the College of Family Physicians of Canada [see Additional Files 1, 2, 3]. They are available to be used by other clinics in English and French [53]. The forms can also be incorporated into an electronic medical record since they are available in an electronic format. Primary care practitioners and health service managers can easily implement this form into clinical practice and be confident that it will improve the delivery of preventive health services.

This trial was different from other reminder systems because it integrated existing practice styles. The single check-list form contains all recommended preventive services for healthy adults as suggested by the Canadian Task Force on Preventive Health Care and other sources, similar to the US Preventive Services Task Force. Many previous reminder forms focused on particular preventive maneuvers such as cancer screening or immunizations. They did not incorporate a broad range of preventive health services, nor existing non-evidence based practice components, such as physical examination and documentation space. Age- and sex-specific prompts were also valuable. No continuing medical education or training session was required to use the forms, though the forms were announced to physicians and medical staff at staff meetings, via email and memos. Forms were placed on the charts of patients scheduled for a check-up by clerical staff. Physicians adopted their own approach to utilize the form. The Preventive Care Checklist Form^© ^is a comprehensive tool not only to deliver preventive health services to healthy adults, but also to act as a documentation tool for the encounter in general. Our hypothesis for why the forms were a success is because the forms provide a rapid way to administer preventive health services that is harmonious to practice, addressing many physicians' concerns that there is "no time for prevention" [29]. In Canada, the Rourke baby record is a widely used checklist for evidence-based pediatric care, so physicians were already familiar with using evidence-based checklist forms [54-57].

No recent studies have demonstrated that checklist reminder sheets alone are still effective for family physicians, especially in today's technological age. Only 5–13% of primary care physicians in the United States have electronic health record systems [58]. Barriers to implement electronic patient care records include costs, varying and untested vendor quality and life expectancy and products that are not tailored to specific practice needs. Small practices may be the last to adopt electronic health records, especially without supports available including financial incentives [59,60]. Seventy-eight percent of physicians in the United States practice in groups of 8 or fewer [58]. Small practices are also less likely to incorporate multi-system interventions to assist in the delivery of preventive health services. The Preventive Care Checklist Form^© ^will be particularly attractive to these small practices. Still, those who have an electronic health record system can incorporate this form into a record of the adult health check-up, as physicians and health record companies in our area have already done so. The forms are available free of charge in a PDF format on-line or can be accessed as a word-processing document [53]. Perhaps the simpler interventions that incorporate existing practice patterns are more effective than complex technological interventions that require training and/or continuing medical education.

Individualization of the forms will also enhance their ability to be implemented into practice. Some physicians/practices may want additional space or to add other recommendations based on their client population. In our study, physicians adopted their own style to use the form. Some physicians completed the form in a single visit. Others completed the history and physical examination in one visit, ordered necessary testing, and brought the patient back in a second visit to go over the results of investigations and provide health habit counseling. Others had a nurse visit with the patient before the physician encounter where the nurse filled in many components of the form. While the form was standardized, its use by physicians was varied. Overall, 77% of physicians who used the form in this intervention said they would continue to use the form in their practice [61]. The commonest reasons for not adopting the forms in routine care were because the layout was thought not to flow easily and to be cluttered, too much repetition, time consuming, or a dislike of using standardized forms [61]. Individualization in the utilization of the form is another reason for its continued success. Individualization has been proven effective in other settings as well and is a successful method to change provider behavior in an acceptable fashion [62,63]. For instance, large staffed health care systems may consider the incorporation of the forms as part of a tool for a pre-visit check by nurses, or further developed and extended based on the goals of the organization.

Much work has been carried out to determine why the implementation of clinical practice guidelines and preventive health guidelines has been so poorly adopted. Numerous interventions and meta-analyses have been conducted to determine which interventions are most successful. Unfortunately, no one intervention or group of interventions has consistently shown to be effective. Even physicians' inclination to provide preventive care is not sufficient as an isolated factor to guarantee that patients will receive preventive health services [45]. Active interventions are more effective than passive interventions and educational outreach and reminders continue to be considered "promising approaches." [8]. When most physicians think of clinical guidelines, on the one hand they think of cookbook medicine which takes away the individualized care plans for patients; on the other hand well developed and evidence-based guidelines can improve patient care [10]. The Preventive Care Checklist Form^© ^is a tool that incorporates the evidence in a way that allows physician flexibility for individual care. Studies have also shown that as physicians age, they are not keeping up with evidence-based approaches to patient care [64]. Having an evidence-based reminder system for physicians can ensure that preventive health services remain up-to-date. Clinicians and clinic mangers also differ in their reasons for using evidence. Clinicians invoke clinical intuition as a guide to most routine clinical decisions, and managers articulate both motivation and interest in using medical research to guide decision-making, most commonly promoted by cost [65]. The Preventive Care Checklist Form^© ^addresses both of these stances.

Some have argued that it may simply be unrealistic to expect primary care clinicians to deliver a comprehensive package of clinical preventive services among the many competing demands of providing primary care, especially since it is suggested that 7.4 hours a day are required to provide only preventive services in primary practice [24,31]. Others have suggested that top ranked preventive services based on burden of disease and cost-effectiveness, such as tobacco counseling or colorectal cancer screening, be implemented with full force over other preventive services [66]. The success of the Preventive Care Checklist Form^© ^to improve the delivery of a wide range of preventive health services refutes these arguments. Others argue that health habit counseling is associated with diminished patient satisfaction. However, in one study, providing tobacco screening and smoking cessation counseling improved patient satisfaction [67].

There are some limitations to consider. Intervention and control patients and physicians were somewhat different in characteristics that may be associated with preventive service delivery. While the groups varied somewhat in physician and patient composition, rates of preventive service delivery were very similar in both groups at baseline. After the intervention, preventive service delivery only changed substantially in the intervention group. As well, controlling for these differences and other potential confounders did not change the main results of the study. Other patient characteristics such as education, occupation, income, language and ethnicity were not available in our setting. Randomization of individual physicians was not appropriate due to the likelihood of contamination between physicians seeing each other's patients at the same clinic. There was no evidence for contamination of the use and/or knowledge of the forms in the control group. Actual use of the forms was limited to the intervention group only and any contamination would bias the results toward the null since it would encourage improved preventive service delivery in the control group. The rates of preventive service delivery did not change in the control group compared to baseline. Apart from the forms we were not aware of any changes in preventive practices or patient population during the five-month study period. This study was conducted at four academic clinics in an urban setting in Canada which may not be generalizable to other settings. Still, the forms are applicable to primary care in general and the baseline rates of preventive maneuvers at our centre are similar to other sites in diverse, non-academic settings [21,24]. Our main outcome measure did not account for preventive measures that were done and not documented or those that were documented and not done, which likely under-represented rates of performance. However, this assumption is consistent with medico-legal standards. Due to age, sex and other eligibility criteria, some preventive health services had small sample sizes, but still many of the results were positive despite decreased power in these sub-strata. We assumed the ICC for primary care outcomes was 0.02 [50]. Given the many statistically significant outcomes, a type II error is minimal. We only looked at preventive maneuvers carried out pre-booked adult health check-ups. A patient who comes in for a complete check-up may not be comparable to a patient who only comes in only for illness visits. Still, acute care needs can be addressed at a complete health check-up, but the focus is on a comprehensive approach. Testing of the form in a broader range of visit types in subsequent work would be helpful since it may be difficult to assure a wellness visit for chronically ill patients.

Some of the post-intervention rates of preventive maneuvers were limited by ceiling effects, such as blood pressure, clinical breast exam, history of smoking and history of alcohol, which all reached rates of over 90%. Of the maneuvers that showed a significant change, there were no consistent patterns favoring health habit counseling, physical examination or procedure/investigation maneuvers, nor were outcomes related to the strength of evidence for the recommendation. Maneuvers that reached statistical significance are not due to documentation alone, since certain services, such as fecal occult blood testing and rubella immunity, have laboratory reports documenting they were performed, rather than just a written check by the physician. These findings suggest that improved delivery of preventive services in the intervention arm is not accounted for by improved documentation alone or preference for maneuvers with more evidence. Still, improved documentation alone will reduce the over delivery of certain services which will in turn free up time for additional services to be delivered [40].

The Task Force and others have recommended the abandonment of the annual health check-up and suggest that preventive health maneuvers be completed at periodic health examinations and others have questioned its necessity [68,69]. However studies have shown that both physicians and patients prefer and are more likely to carry out preventive health maneuvers at routine well visits [68-71]. A complete health check-up is a chance to build trust with the patient, to be thorough, and to use it as an organizational strategy to offer preventive health care. Patients are also more likely to recall advice during a well care visit such as a health check-up than during an illness visit [72].

## Conclusion

The Preventive Care Checklist Form^© ^is an evidence-based, validated, simple, low cost, clinically relevant tool [see Additional Files 1, 2, 3]. It facilitates uptake into practice in an attractive way by making the delivery of preventive health care easier rather than more difficult. It prompts physicians about issues that are difficult to remember, especially since many maneuvers are age and sex-specific. It incorporates requirements from insurance payers and practice patterns that are not evidence-based, but a part of routine care. In our setting, no documentation other than the form is required for the visit. The colourful form is easy to find in a chart and facilitates documentation of ongoing preventive care. It is easy to incorporate into practice and is inexpensive. It does not require computer systems, nurse facilitators or patient materials, yet it can be readily incorporated into an electronic medical record system. Periodic updates of the form will keep preventive primary care current with evolving evidence, and ease the delivery of preventive services.

## Competing interests

The author(s) declare that they have no competing interests.

## Authors' contributions

VD, RM1, KI developed the Preventive Care Checklist Forms^©^. All authors were involved in study design. VD, RM1, KI were involved in implementing the study. RM2 conducted the statistical analysis. VD, RG wrote the paper and oversaw the study.

## Pre-publication history

The pre-publication history for this paper can be accessed here:



## Supplementary Material

Additional File 1 **Male Preventive Care Checklist Form**. This is the Male Preventive Care Checklist Form that was used in the trial, in a pdf format. Click here for file

Additional File 2 **Female Preventive Care Checklist Form**. This is the Female Preventive Care Checklist Form that was used in the trial, in a pdf format. Click here for file

Additional File 3 **Preventive Care Checklist Form Explanations**. This is the evidence-based explanation sheet that accompanied the Preventive Care Checklist Forms in the trial which detailed the evidence for each preventive health maneuver (in a pdf format). Click here for file
