# Supplementary material for: Improving preventive service delivery at adult complete health check-ups: the Preventive health Evidence-based Recommendation Form (PERFORM) cluster randomized controlled trial
Source: BMC Fam Pract. 2006 Jul 12;7:44. doi: 10.1186/1471-2296-7-44 (PMC1543627; doi:10.1186/1471-2296-7-44)
Supplement: Additional File 2 — Female Preventive Care Checklist Form. This is the Female Preventive Care Checklist Form that was used in the trial, in a pdf format. [file 1471-2296-7-44-S2.pdf]

Preventive Care Checklist Form©

For average-risk, routine, female health assessments

Developed by: Drs. V. Dubey, R. Mathew, K. Iglar

Please note:

Bold = Good evidence (from the Canadian Task Force on Preventive HealthCare)
Italics = Fair evidence (from the Canadian Task Force on Preventive HealthCare)
Plain text = Guidelines (from other Canadian sources)
(See reverse for references, insert for explanations)

Name: Sex:

DOB: Age:

Health Card: Tel:

Address:

Date:

Current Concerns

Lifestyle/Habits

DIET:
Fat / Cholesterol
Fiber
Calcium
Sodium

EXERCISE:

WORK:

FAMILY:

RELATIONSHIPS:

SMOKING:

ALCOHOL:

DRUGS:

SEXUAL HISTORY:

FAMILY PLANNING/ CONTRACEPTION:

SLEEP:

Update Cumulative Patient Profile

O Family History
O Hospitalizations/ Surgeries
O Medications
O Allergies

Functional Inquiry

	NORMAL	REMARKS
HEENT:	O	
CVS:	O	
RESP:	O	
BREASTS:	O	
GI:	O	
GU/ MENSES:	O	
SEXUAL FUNCTION:	O	
MSK:	O	
NEURO:	O	
DERM:	O	
MENTAL HEALTH:	O	
CONSTITU-TIONAL SX:	O	

Education/ Counseling

For general population unless otherwise stated

Behavioural

O **folic acid** (0.4-0.8 mg OD, for childbearing women)
O *adverse nutritional habits*
O adequate calcium intake (1000 to 1500mg/d)[1]
O adequate vitamin D (200 IU in 50-64, 400-800 IU in ≥65 yr)[1]
O *regular, moderate physical activity*
O *avoid sun exposure, use protective clothing*
O *safe sex practices/STD counseling (esp gonorrhea)*
O *pros/cons of HRT (perimenopausal/menopausal)*

Smoking O Yes O No

O **smoking cessation**
O **nicotine replacement therapy**
O *dietary advice on fruits and green leafy vegetables*
O *referral to validated smoking cessation program*

Alcohol O Yes O No

O *case finding for problem drinking*
O *counseling for problem drinking*

Elderly O Yes O No

O ***cognitive assessment*** (if concerns)
O **fall assessment** (if history of falls)

Oral Hygiene

O ***brushing/flossing teeth***
O **fluoride (toothpaste/supplement)**
O *tooth scaling and prophylaxis*
O ***smoking cessation***

Personal Safety

O **hearing protection**
O **noise control programs**
O *seat belts*

Parents with children

O Yes O No
O *poison control prevention*
O *smoke detectors*
O *non-flammable sleepwear*
O *hot water thermostat settings*

Please note:
Bold = Good evidence (from the Canadian Task Force on Preventive HealthCare)
Italics = Fair evidence (from the Canadian Task Force on Preventive HealthCare)
Plain text = Guidelines (from other Canadian sources)
(See reverse for references, insert for explanations)

Disclaimer: This form is a guide to the adult periodic health examination. Last updated June 2004. The recommendations are for average-risk adults.

Endorsed by: The College of Family Physicians of Canada / Le Collège des médecins de famille du Canada

Name:

Physical Examination	
HR: *BP*: RR:	HT: WT: BMI:
EYES: *Snellen sight card: R* *L*	**BREASTS**:
NOSE:	ABDO:
EARS: *whispered voice test: R* *L*	ANO-RECTUM:
MOUTH/THROAT:	PELVIC: O *Pap*
NECK/THYROID:	NEURO:
CVS:	DERM:
RESP:	MSK/JOINTS/ EXTREMITIES:

Age	21-64 years	≥65 years
Labs/ Investi-gations	O **Mammography** (50-69 years; q1-2 yrs)<br>O **Hemoccult multiphase q1-2 years** (age ≥ 50)<br>OR O *Sigmoidoscopy*<br>O *Cervical Cytology q1-3 yrs* (sexually active until age 69)<br>O **Gonorrhea**/ *Chlamydia*/ **Syphilis screen** (high risk)<br>O Fasting Lipid Profile (≥50 yr or postmenopausal or sooner if at risk)[2]<br>O Fasting Blood Glucose, at least q3 yrs (≥40 yr or sooner if at risk) [3]<br>O Bone Mineral Density if at risk[1]	O **Mammography** (until age 69; q1-2 yrs)<br>O **Hemoccult Multiphase q1-2 years**<br>OR O *Sigmoidoscopy*<br>O *Audioscope (or inquire/whispered voice test)*<br>O Fasting Lipid Profile[2]<br>O Fasting Blood Glucose, at least q3 yrs (more often if at risk) [3]<br>O Bone Mineral Density q1-2 years if abnormal, q2-3 years if normal[1]
Immuni-zations	O **Tetanus vaccine q10yr**<br>O *Rubella vaccine* O *Rubella Immunity*<br>O *Varicella vaccine (2 doses)* O *Varicella Immunity*<br>O Pneumococcal vaccine (high risk) [4]<br>O Influenza vaccine q1yr (patient request or high risk) [4]	O **Influenza vaccine q1yr**<br>O **Tetanus vaccine q10yr**<br>O *Varicella vaccine (2 doses)* O *Varicella Immunity*<br>O Pneumococcal vaccine[4]

Assessment and Plans:

Date: **Signature:**

References

Unless otherwise stated, recommendations come from the Canadian Task Force on Preventive Health Care: *The Canadian Guide to Clinical Preventive Health Care*. Ottawa: Minister of Supply and Services Canada and http://www.ctfphc.org/

1. Scientific Advisory Board, Osteoporosis Society of Canada. Clinical practice guidelines for the diagnosis and management of osteoporosis. *CMAJ* 2002;167(10 suppl):S1-34.
2. Working Group on Hypercholesterolemia and Other Dyslipidemias. Recommendations for the management and treatment of dyslipidemia and the prevention of cardiovascular disease: 2003 update. *CMAJ online* 2003;169(9) 1-10.
3. Canadian Diabetes Association Clinical Practice Guidelines Expert Committee. Canadian Diabetes Assn 2003 Clinical Practice Guidelines for the Prevention and Management of Diabetes in Canada. *Can J Diabetes.* 2003;27 (Suppl 2).
4. National Advisory Committee on Immunization. *Canadian Immunization Guide*, 6th edition. Ottawa: Minister of Public Works and Government Services Canada; 2002.

Please note:
Bold = Good evidence (from the Canadian Task Force on Preventive HealthCare)
Italics = Fair evidence (from the Canadian Task Force on Preventive HealthCare)
Plain text = Guidelines (from other Canadian sources)

Disclaimer: This form is a guide to the adult periodic health examination. Last updated June 2004. The recommendations are for average-risk adults.

Endorsed by:
The College of Family Physicians of Canada
Le Collège des médecins de famille du Canada
