# Supplementary material for: Improving preventive service delivery at adult complete health check-ups: the Preventive health Evidence-based Recommendation Form (PERFORM) cluster randomized controlled trial
Source: BMC Fam Pract. 2006 Jul 12;7:44. doi: 10.1186/1471-2296-7-44 (PMC1543627; doi:10.1186/1471-2296-7-44)
Supplement: Additional File 3 — Preventive Care Checklist Form Explanations. This is the evidence-based explanation sheet that accompanied the Preventive Care Checklist Forms in the trial which detailed the evidence for each preventive health maneuver (in a pdf format). [file 1471-2296-7-44-S3.pdf]

Explanations for Preventive Care Checklist Form© (Page 1/2)

Education/Counseling

Behavioural

Folic acid (A):

- To prevent neural tube defects (NTD) in all women capable of becoming pregnant
- Low risk-women: Folic acid supplementation of 0.4 to 0.8 mg daily taken at least one month before and three months after conception.
- High-risk women (previous pregnancy with NTD): Supplementation with 4 mg folic acid daily during 3 months before and 3 months after conception reduces recurrence.

Adverse nutritional habits (B):

- Prevention of coronary artery disease, colon cancer
- Provide general dietary advice: decrease fat, increase fiber.
- Those at increased risk, consider referral to a clinical nutritionist or other professional with specialized nutritional expertise.

Dietary advice on fat/cholesterol (B):

- Prevent coronary heart disease
- Evidence is only for men aged 30-69.
- Decrease intake of total fat, saturated fat, and cholesterol

Calcium 1000-1500mg/day[2]:

- Prevent osteoporosis
- The Osteoporosis Society of Canada (OSC) recommends adults have 1000 to 1500 mg of elemental calcium per day.
- If this amount cannot be provided by diet alone (for most, three or more servings of dairy products), then calcium supplementation should be recommended.[2]

Vitamin D[2]:

- Prevent osteoporosis
- The OSC recommends 200 IU/day in 50-64 year olds
- 400-800 IU/ day in people ≥ 65 years or with osteoporosis

Moderate physical activity (B):

- Prevention of cardiovascular disease and hypertension.
- Physical activity can also contribute to the prevention of obesity, Type II diabetes mellitus and osteoporosis.
- Recommend moderate-level physical activity performed consistently to accumulate 30 minutes or more over the course of most days of the week.
- Moderate intensity physical activities include: normal walking, golfing on foot, slow biking, raking leaves, cleaning windows, slow dancing, light restaurant work.
- Note: Doing moderate physical activity is a B recommendation but physician counseling is a C.

Avoid sun exposure, use protective clothing (B):

- Prevent skin cancer
- Evidence from epidemiologic studies focusing on etiology of melanoma, prudence and low cost/side-effects, supports the avoidance of excessive sun exposure at mid-day, plus the use of protective clothing.
- Sunscreen use is a C recommendation for general population (last updated 1994).

Safe sex practices/ STD counseling (esp. Gonorrhea counseling) (B):

- Prevent transmission of sexually transmitted diseases.
- Abstinence is most effective, fair evidence to use condoms.
- Counseling and educational materials result in no increase in compliance with medications or willingness to inform sexual contacts, but increase patient compliance with follow-up.

Counseling on HRT (perimenopausal/menopausal) (B):

- Prevent osteoporosis
- The benefits of estrogen replacement therapy must be weighed against its risks.

Smoking Counseling • To Prevent Tobacco-Caused Disease

- **Smoking cessation (A):** counseling effective in reducing the proportion of smokers.
- **Nicotine replacement therapy (A):** may be offered as an adjunct to smoking cessation. It increases cessation rates.
- *Fruit and Green leafy vegetables for smokers:* eat an average of seven portions of green leafy vegetables or fruit per week to lower risk of lung cancer.
- *Referral to validated smoking cessation program:* Referral by physician improves participation in group programs.

Alcohol Counseling

- Prevent Alcohol related Morbidities
- *Case finding for problem drinking (B):* Standardized questionnaires (e.g. CAGE, AUDIT) and/or patient inquiry.
- *Counseling for problem drinking (B):* Clarify association between alcohol consumption and alcohol-related consequences, advice to reduce consumption.

Elderly

- ***Cognitive assessment (A and B)***:
- When caregivers or informants describe cognitive decline in an individual, these observations should be taken very seriously; cognitive assessment and careful follow-up are indicated (A).
- Memory complaints by patient or caregiver should be evaluated and the individual followed to assess progression (B).
- **Fall assessment:**
- Good evidence to perform multidisciplinary post-fall assessment on elderly patients who have a history of falls or referring elderly patients to multidisciplinary post-fall assessment teams, where such a service is available (A).
- There is insufficient evidence to support including assessment and counseling of elderly patients for the risk of falling in the routine health exam of the elderly (C).

Oral Hygiene • To prevent periodontal disease, and oral cancer

- ***Brushing/flossing teeth (A, B):*** Flossing teeth is effective in preventing gingivitis in adults. Brushing teeth is essential in the application of fluoride dentifrice to prevent dental caries (A) and prevention of gingivitis (B).
- **Fluoride (toothpaste/supplement) (A):** Daily use of fluoride toothpaste gives significant reductions in decay and/or daily fluoride supplements (only where water fluoride levels are less than optimal).
- *Tooth scaling and prophylaxis (B):* In periodontally healthy patients, intensive professional oral hygiene and prophylaxis prevents chronic gingivitis and periodontitis. Annual scaling provides no additional benefit for those who maintain good oral hygiene.
- ***Smoking cessation (A, B):*** To reduce the risk of oral cancer. Intervention programs have reduced the incidence of precancerous lesions. Also prevents periodontal disease due to smoking (B).

Personal Safety

- *Seat belts (B)*: Prevent injury from Motor Vehicle Collisions. Physicians can influence significant short-term improvement in seat belt use.
- **Noise control programs/hearing protection (A)**: Good evidence to support noise control programs and hearing protection but no comment made on impact of physician counseling.

Parents with children < 15 years of age

- *Poison control prevention (B)*: Counseling on prevention of poisoning and poison control centre phone number stickers to the parents of young children.
- *Smoke detectors, non-flammable sleepwear and hot water thermostat settings (B):* Counseling can increase the number of safety features in the home but impact on injury is unknown.

References

Unless otherwise stated, recommendations come from 1. The Canadian Task Force on Preventive Health Care: *The Canadian Guide to Clinical Preventive Health Care*. Ottawa: Minister of Supply and Services Canada and http://www.ctfphc.org/

1. Scientific Advisory Board, Osteoporosis Society of Canada. Clinical practice guidelines for the diagnosis and management of osteoporosis. *CMAJ* 2002;167(10 suppl):S1-34.
2. Working Group on Hypercholesterolemia and Other Dyslipidemias. Recommendations for the management and treatment of dyslipidemia and the prevention of cardiovascular disease: 2003 update. *CMAJ online* 2003;169(9) 1-10.
3. Canadian Diabetes Association Clinical Practice Guidelines Expert Committee. Canadian Diabetes Assn 2003 Clinical Practice Guidelines for the Prevention and Management of Diabetes in Canada. *Can J Diabetes.* 2003;27 (Suppl 2).
4. National Advisory Committee on Immunization. *Canadian Immunization Guide*, 6th edition. Ottawa: Minister of Public Works and Government Services Canada; 2002.

Please note:

Bold = Good evidence (from the Canadian Task Force on Preventive HealthCare)

Italics = Fair evidence (from the Canadian Task Force on Preventive HealthCare)

Plain text = Guidelines (from other Canadian sources)

Endorsed by: The College of Family Physicians of Canada / Le Collège des médecins de famille du Canada

Explanations for Preventive Care Checklist Form © (Page 2/2)

Physical Examination

Blood Pressure(B): To screen for hypertension.
- If BP is mildly elevated do three further readings over 6 months.
- If BP severely elevated, shorten intervals between readings.
- Treatment of hypertension in adults lowers risk of stroke, cardiac events and death (A recommendation).

BMI (Body Mass Index):
- B recommendation for obese adults with obesity-related disease.
- C recommendation for general adult population.

Screening for hearing impairment in elderly (B):
- All of the following have high sensitivity to detect hearing loss
- *Whispered voice test:* Whispered-voice, out of field of vision
- *Audioscope*
- *Inquiry:* A single question to ask about hearing difficulty

Snellen test (B):
- In elderly, reliably detects reduced visual acuity.

Breasts (A): • To screen for breast cancer.
- Screen women aged 50-69 years by clinical examination and mammography, every 1-2 years.
- See investigations/labs section for Mammography explanations.

Pap(B): • To screen for invasive cervical carcinoma.
- Annual screening following initiation of sexual activity or age 18 (sic)
- After 2 normal smears, screen every 3 years to age 69.
- Increase frequency for women with risk factors: age of first sexual intercourse < 18 yrs, many sexual partners or consort with many partners, smoking or low socioeconomic status.

Immunizations

Tetanus (A):
- Routine booster doses every 10 years if had primary series
- Adults without a primary series need three doses.
- Primary adult series are given at time 0, 1-2 months, and 6-12 months.[5]

Rubella (B): According to NACI[5]:
- Give one dose of a vaccine to non-pregnant women of childbearing age unless there is proof of immunity via immunization records or serology.
- They do not recommend serologic testing as it may delay needed immunization.[5]
- According to the Task Force, the decision on universal vaccination or screening for immunization status followed by vaccination depends on the physician's practice setting (i.e., cost of serologic testing, will patient follow-up for needed vaccination).[1]

Varicella (B):
- Determine Varicella infection status by history or serology.
- Adminster to those susceptible to Varicella infection, including: women of childbearing age (but not during pregnancy), health care workers, household contacts of immunocompromised people, adults who may be exposed at work (teachers, day care workers), other susceptible adults, especially new immigrants from tropical climates.
- Give two doses at least 4 weeks apart, if ≥ 13 years.[5]

Pneumococcal vaccine (A)[1, 5]**:** • To all persons ≥ 65 years
- High Risk: All persons >5 years with the following conditions: sickle cell disease, asplenia, splenic dysfunction, chronic cardiorespiratory disease (except asthma), cirrhosis, alcoholism, chronic renal disease, nephrotic syndrome, diabetes mellitus, chronic CSF leak, HIV infection, smokers, and other conditions associated with immunosuppression (Hodgkin's disease, lymphoma, multiple myeloma, induced immunosuppression for organ transplantation).

Influenza vaccine (A)[1, 5]**:** • Annually immunize the following:
- ≥ 65 years of age
- Adults and children with chronic cardiac or pulmonary disorders (including bronchopulmonary dysplasia, cystic fibrosis, and asthma) severe enough that they have regular follow-up and hospital care,
- Adults and children with chronic diseases, eg. diabetes mellitus, metabolic diseases, cancer, immunodeficiency, immunosuppression, renal disease, anemia, and hemoglobinopathy,
- People at high risk of influenza complications who are traveling to places where the virus is likely present,
- Health care workers and other personnel who have significant contact with people in the high-risk groups previously described
- Household contacts (including children) of a high risk person who cannot be immunized or may respond inadequately to immunization.

Investigations/Labs

Mammography with Clinical Breast Exam (A):
- Screen women aged 50-69 every 1-2 years for breast cancer.
- C recommendation for women aged 40-49 at average risk.
- Frequent screening may be required (every 12-18 months) in 40-49 year group.
- Upon reaching the age of 40, women should be informed of the potential benefits and risks of screening mammography to decide at what age they wish to begin screening.

Colorectal cancer screening of patients ≥ 50 years:
- **Hemoccult multiphase every 1-2 years (A)** OR
- *Flexible Sigmoidoscopy (B)* (Frequency not established)

Cervical cytology (B): • See Pap explanation.

Screening for Sexually Transmitted Diseases in High Risk Populations:
- High-risk Populations: individuals under age 30 with at least 2 sexual partners in the previous year or age 16 years at first intercourse, prostitutes, sexual contacts of individuals known to have an STD.
- **Syphilis (A)**: Serology testing
- **Gonorrhea (A):** Screen with Gram stain and culture of cervical or urethral specimen. Urine testing available at some centers.
- *Chlamydia (B)*: Screen with culture or polymerase chain reaction (PCR) for all sites; direct fluorescent antibody (DFA) for genitourinary (GU), conjunctival (CJ), rectal and nasopharyngeal sites; enzyme-linked immuno-assays (EIA) for GU or CJ specimens; DNA probes for GU specimens. Urine testing available at some centers.

Bone Mineral Density: screen if 1 major or 2 minor risk factor(s)

Major risk factors: age > 65, vertebral compression fracture, fragility fracture after age 40, family history of osteoporotic fracture, systemic glucocorticoid therapy of >3 months duration, malabsorption syndrome, primary hyperparathyroidism, propensity to fall, osteopenia apparent on x-ray film, hypogonadism, early menopause (< 45 years)

Minor Risk Factors: rheumatoid arthritis, past history of clinical hyperthyroidism, chronic anticonvulsant therapy, low dietary calcium intake, smoker, excessive alcohol intake, excessive caffeine intake, weight <57 kg, weight loss >10% of weight at age 25, chronic heparin therapy

Fasting Lipid Profile (total cholesterol, HDL-C, triglyceride and LDL-C)[3]:
- Screen all men over the age of 40, women who are postmenopausal or over the age of 50
- Screen adults with 1 or more risk factors for CAD (eg. hypertension, smoking, abdominal obesity); patients with clinical evidence of CAD; peripheral vascular disease or carotid atherosclerosis; patients with diabetes mellitus; patients with xanthomata or other stigmata of dyslipidemia; and patients with a family history of dyslipidemia or CAD.
- Optimal frequency for screening is unknown. For asymptomatic patients, reasonable to screen every 5 years or sooner if new risk factors are acquired.

Target Lipid Values by Level of Risk[3]

Level of Risk	LDL-C level mmol/L		Total Cholesterol to HDL-C ratio
**High** (10 yr risk of CAD ≥20% or hx of CHD or DM)	<2.5	**and**	<4.0
**Moderate** (10 yr risk 11-19%)	<3.5	**and**	<5.0
**Low** (10 yr risk ≤10%)	<4.5	**and**	<6.0

Fasting plasma glucose (FPG)[4]: • Screen for Type II diabetes mellitus
- Should be done every 3 years in adults ≥40 years of age or more frequently if one of the following risk factors:

· First-degree relative with DM<br>· Presence of complications associated with DM<br>· Hypertension<br>· Dyslipidemia<br>· Overweight	· Member of high risk population (Aboriginal, Hispanic, Asian, South Asian or African descent)<br>· History of impaired glucose tolerance or impaired fasting glucose<br>· Abdominal obesity	· Vascular disease<br>· History of gestational DM or macrosomic infant<br>· Schizophrenia<br>· Polycystic ovarian syndrome<br>· Other risk factors

- FPG ≥ 7.0 mmol/L is diagnostic of diabetes mellitus

Please note:
Bold = Good evidence (from the Canadian Task Force on Preventive HealthCare)
Italics = Fair evidence (from the Canadian Task Force on Preventive HealthCare)
Plain text = Guidelines (from other Canadian sources)

Endorsed by:
The College of Family Physicians of Canada — Le Collège des médecins de famille du Canada
